# Ultrasound-guided versus Shikani optical stylet-aided tracheal intubation: a prospective randomized study

**DOI:** 10.1186/s12871-020-01133-4

**Published:** 2020-09-03

**Authors:** Yuanyuan Ma, Yan Wang, Ping Shi, Xue Cao, Shengjin Ge

**Affiliations:** 1 Department of Anesthesia, Zhongshan Hospital, Fudan University, No. 180 Fenglin Road, Shanghai, 200032 China; 2 Kashgar Regional Second People’s Hospital, Kashi City, Xinjiang Uygur Autonomous Region China

**Keywords:** Ultrasound, Shikani optical Stylet, Tracheal intubation, Anesthesia

## Abstract

**Background:**

To compare ultrasound-guided tracheal intubation (UGTI) versus Shikani optical stylet (SOS)-aided tracheal intubation in patients with anticipated normal airway.

**Methods:**

Sixty patients aged 18–65 years old who presented for elective surgery under general anesthesia were recruited in this prospective randomized study. They were assigned into two equal groups, either an ultrasound-guided group (Group UG, *n* = 30) or an SOS-aided group (Group SOS, *n* = 30). After the induction of anesthesia, the tracheal intubation was performed by a specified skilled anesthesiologist. The number of tracheal intubation attempt and the duration of successful intubation on the first attempt were recorded. Complications relative to tracheal intubation including desaturation, hoarseness and sore throat were also recorded.

**Results:**

The first-attempt success rate is 93.3% (28/30) in Group UG and 90% (27/30) in Group SOS (*P* = 0.640). The second-attempt was all successful for the 2 and 3 patients left in the two groups, and the overall success rate of both groups was 100%. The duration of successful intubation on the first attempt of Group UG was not significantly different from that of Group SOS (34.0 ± 20.8 s vs 35.5 ± 23.2 s, *P* = 0.784). One patient in Group SOS had desaturation (*P* = 0.313), and there was none hoarseness in the two groups. Sore throat was detected in both group (4 in Group UG, 5 in Group SOS, *P =* 0.718).

**Conclusion:**

Ultrasound-guided tracheal intubation was as effective as Shikani optical stylet-aided tracheal intubation in adult patients with anticipated normal airway.

**Trial registration:**

Chinese Clinical Trial Registry, ChiCTR-IIC-17010875. Date of Registration: 15 March 2017.

## Background

Nowadays, ultrasound is used to manage the airway in anesthesia and intensive care as a non-invasive tool. Real-time surface ultrasound-guided tracheal intubation (UGTI) was firstly reported by Fiadjoe et al. [1] on a 14-month-old child. They provided a new perspective of endotracheal intubation: from the outside rather than the inside. Optical stylet is also a nonconventional intubation tool, which integrates flexible fiberoptic imaging features in a rigid intubating stylet. The Shikani optical stylet (SOS) (Clarus Medical, Minneapolis, MN) is one of the most commonly used fiberoptic stylets for intubation. It not only has the characteristics of a fiberoptic bronchoscope but also very light and convenient [2, 3]. UGTI and SOS-aided tracheal intubation have some similar manipulations in the intubation: opening the mouth with hands, placing the styletted tube, and inserting the tube with the guidance of ultrasound or light spot and optical stylet. And they are particularly effective for some special patients, who has limitation of mouth opening or difficult visualization of the airway obscured by secretions or blood.

The purpose of this study was to compare UGTI with SOS-aided tracheal intubation in patients with anticipated normal airway undergoing elective surgery under general anesthesia.

## Methods

### Ethics approval and registration

This prospective randomized study was approved by the Ethical Committee of Zhongshan Hospital, Fudan University (Approval No: B2016-099R). The study was registered at chictr.org.cn (ChiCTR-IIC-17010875). Written informed consent was obtained from all participants.

### Patient population

This study adhered to CONSORT guidelines for randomized trials. Sixty patients aged 18–65 years old who presented for elective surgery under general anesthesia were recruited at Zhongshan Hospital, Fudan University. Inclusion criteria included a body mass index (BMI) of 18.5–25 kg·m^− 2^, American Society of Anesthesiologists (ASA) physical status I-II, and Mallampati Grade I-II. Exclusion criteria included any sign of difficult airway such as mouth and neck mass or infection, allergy to the study drugs, drug or alcohol abuse, and pregnancy.

### Anesthetic technique

Using a computer-generated table and a sealed envelope with sequence of numbers, patients were randomly divided into two equal groups: an ultrasound-guided group (Group UG, *n* = 30) and an SOS group (Group SOS, *n* = 30).

A specified attending anesthesiologist conducted the pre-anesthetic interview to the patients. All participants were fasting according to the rules of Zhongshan Hospital without preoperative medications. After patients arrived at the operating room, an 18G peripheral venous catheter was established, and oxygen (8 L·min^− 1^) was supplemented via face mask. Intraoperative monitoring included pulse oxygen saturation (SpO_2_), electrocardiogram (lead II and V_5_), heart rate, noninvasive blood pressure, and capnography.

After pure oxygen with a flow rate of 8 L·min^− 1^ was inhaled for 3 min via a sealed mask, plasma target-controlled infusion of propofol 4 μg·ml^− 1^, intravenous fentanyl 2 μg·kg^− 1^, remifentanil 0.2 μg·kg^− 1^·min^− 1^ and dexamethasone 5 mg were given. After loss of consciousness, rocuronium 0.6 mg·kg^− 1^ and lidocaine 1.5 mg·kg^− 1^ were administered. Ninety seconds later, the tracheal intubation was carried out according to the randomly allocation by a specified anesthesiologist who had performed UGTI or SOS-aided tracheal intubation for more than 50 times, respectively.

Before the manipulation of tracheal intubation in the two groups, the ready-to-use stylet was lubricated and bent to imitate the shape and structure of the airway from the central incisors to the cricoid cartilage, then an endotracheal tube (7.0 mm ID for female, 7.5 mm ID for male) was mounted on it.

#### Group UG

A 6–13 MHz linear ultrasound probe (Sonosite EDGE ultrasound) was put transversely on the patient’s neck at the level of the cricothyroid membrane to guide intubation. The probe cloud been moved cephalad to obtain a clear image of the vocal cords that were shown as an isosceles triangle with a central tracheal shadow. The specified anesthesiologist opened patient’s mouth and maintained manual inline stabilization and jaw thrust while inserting the pre-shaped styletted tracheal tube. The tube should be kept in the midline of the mouth during the operation. Hypoechoic shadowing and widening of the vocal cords were the certification of successful placement of the tube into the trachea. When the tube was to be stuck in the midline using the ultrasound, then it was withdrawn outside from the mouth and reshaped by bending its tip downwards. Once the tracheal tube passed posterolateral of the airway to the esophagus which was a hyperechoic structure with posterior shadowing in the image of ultrasound, it should be withdrawn. Then, the tip of tube required to be bent upwards and then reinserted [1, 4].

#### Group SOS

The specified anesthesiologist held and elevated the mandible using the left hand; the stylet was introduced from the right side of the mouth. Thereafter, a significant light spot was seen through the front of neck, the tip was inserted into the glottis under direct vision, the “tube stop” was released. The tube was uninstalled into the trachea, then the stylet was removed.

After the correct position of the tube was confirmed by the end-tidal of carbon dioxide (ETCO_2_) monitoring and stethoscope, the anesthesia machine was connected and parameters were set to maintain the partial pressure of ETCO_2_ at 30–40 mmHg. The failure of intubation attempt defined as the time > 180 s or desaturation (SpO_2_ < 93%). To ensure patient safety, the intubation attempt must be stopped and ventilated with pure oxygen for 3 min to perform another attempt. Failure of intubation was considered as 2 failed attempts of insertion, then intubation was executed with a ready video laryngoscope.

The time of the intubation was from passage of the mounted tracheal tube between the teeth until the tracheal tube was confirmed in place by the capnography, and the number of tracheal intubation attempt were recorded. Complications relative to tracheal intubation including desaturation, hoarseness and sore throat were also recorded.

### Data collection

Our null hypothesis was: the success rate of UGTI at the first attempt is equal to that of SOS-aided tracheal intubation in patients with anticipated normal airway undergoing elective surgery under general anesthesia. Then, the primary outcome was to compare the success rate of intubation of the two groups on the first attempt. The secondary outcomes were to detect the overall success rate, the time of the intubation with a first-attempt success, and complications related to each method, such as desaturation and trauma.

The sample size was calculated using the PASS software version 11: Two-Sample T-Test Power Analysis. It was estimated that a sample size of 34 patients (*n*_*1*_ = *n*_*2*_ = 17) would achieve a power of 80% (β = 0.2) to detect a clinical significant difference of 5% with standard deviations of 5% between the two groups as regards the success rates at the first attempt using a T-Test with significance level at 0.05 (α = 0.05).

### Statistical analysis

Data were analyzed by using IBM SPSS version 24 software. Qualitative data were described using number and were compared using the Chi-square test and the Fisher exact test, whereas normally distributed quantitative data were expressed as mean ± SD and were compared using T*-*Test for two independent groups, non-normally distributed quantitative data were expressed as median (Inter-Quartile Range) and were compared using Mann-Whitney U test. *P* < 0.05 was considered statistically significant.

## Results

Demographic data in both groups are presented in Table 1. There was no statistically significant difference between the two groups about demographics including sex, age, height, weigh (*P* **=** 0.301, 0.273, 0.835, and 0.726, respectively).

**Table 1:** Demographic data in the two groups

Group	n	M/F	Age (years old)	Height (cm)	Weight (kg)	Mallampati Grade (I / II)
UG	30	16/14	49.3 ± 11.3	164.7 ± 8.6	63.6 ± 10.8	15/15
SOS	30	12/18	45.8 ± 13.1	164.3 ± 7.5	62.6 ± 11.2	15/15

The first-attempt success rate is 28/30 in Group UG and is 27/30 in Group SOS (*P* = 0.640). The reason of the failure at the first-attempt in both 2 patients in the two groups was taking time > 180 s, and in another 1 patient in Group SOS was SpO_2_ < 93%. The second-attempts were all successful for the 2 and 3 patients left in the two groups (*P* = 0.640), then the overall success rate of both groups was 100%. Comparing the time of the intubation with a first-attempt success between the two groups, there was no significant difference (34.0 ± 20.8 s in Group UG and 35.5 ± 23.2 s in Group SOS, respectively) with a *P* value of 0.784.

All patients successfully underwent the intraoperative period and were extubated uneventfully at the end of the operations. One patient in Group SOS had desaturation (*P* = 0.313), none hoarseness happened, and sore throat was observed in 4 patients (13.3%) in Group UG and 5 (16.7%) in Group SOS (*P* = 0.718).

## Discussion

At present, there are many kinds of endotracheal intubation assistant tools, the most used are video laryngoscope (bladed laryngoscope) and ordinary direct laryngoscope. In the treatment of difficult airway, the flexible fiberoptic bronchoscope is the classical and universally accepted choice. Optical stylets integrate flexible fiberoptic imaging features in a rigid intubating stylet. Not only can it be applied to normal airway intubation, but also shows the prospect of assisting difficult intubations. The SOS, one of the most used intubating fiberoptic stylets, has the unique advantages and characteristics. Firstly, it has a portable and reusable scope with a shapeable stainless-steel stylet and adjustable tube stop. Secondly, a special port allowed to delivery oxygen to the patient. Furthermore, the high-resolution eyepiece with light source can be used independently, or connected with a camera or a monitor. It has been widely used in clinic because of its fast, effective and little effect on hemodynamics [2, 3].

Ultrasound technology is more and more used in clinical anesthesia due to its safe, reliable, repeatable and easy to realize characteristics. In the case of endotracheal intubation, using ultrasound image, we can determine the endotracheal tube size [5–7], observe the position of cricothyroid membrane and glottis [1, 4, 5, 8], guide tracheal intubation [1, 4, 5], judge whether the tracheal tube enters the trachea [1, 4, 5, 9], and judge whether the position of tracheal tube is correct [5, 10–12]. At present, portable ultrasound is the standard equipment in many hospitals, which is easy to get, and is very suitable for promotion and application in basic hospitals.

UGTI is still a relatively new method in China and aboard. As mentioned above, real-time surface UGTI and SOS-aided tracheal intubation are similar in their operational procedures and applicable patients. To our knowledge, there is no study comparing these two methods. The purpose of this study is to compare UGTI with SOS-aided tracheal intubation, to explore the feasibility and practicability of UGTI, and to provide reference for clinical airway management. As the results showed, both ultrasound and SOS were successfully applied to endotracheal intubation with comparable first-attempt success rate and time for tracheal intubation.

In this study, we selected the anesthetized adult patients with normal airways to perform UGTI. We found that the tracheal tube and stylet were not hyperechoic, but their characteristic hypoechoic shadow in the pharynx could be immediately identified and then guided into the trachea with ultrasonography. The characteristic of UGTI is to be guided by the external view of the airway rather than the internal visualization of the airway. What’s more, the operator could evaluate the relationship between the tracheal tube tube with the glottic structures in the whole process of intubation. The visual information of ultrasound could assist to identify the location of the resistance either on the arytenoid cartilages or the vocal cords during advancing the styletted tube. Then, the operator could make some adjustments to bypass the resistance [1, 4, 5].

There are some limitations of UGTI. Firstly, two anesthesiologists are required to complete UGTI, one of them to obtain ultrasound visualization and the other to intubate. Secondly, the poor ultrasound images might affect its application. Thirdly, it is necessary for the operator to have the basic knowledge of ultrasound machine in order to perform the typical controls and adjustments. Notably, it is essential to conduct clinical training in visualization of the airway structures and sonographic features of successful tracheal intubation using ultrasound before attempting UGTI [4]. Fourthly, further evaluation is required to determine the optimal stylet structure, although the stylet has been bent to simulate the shape of airway from the central incisors to the cricoid cartilage. Fifthly, this technique has the potential for airway injury, although accurate the ultrasound image was necessary for correct intubation. Regardless of the intubation method, the anesthesiologist should stop inserting the tracheal tube once resistance appeared. Inappropriate strength might increase the risk of airway injury during the procedure of intubation. Sixthly, it is worth mentioning that experience and capability are critical to the successful of any techniques in clinical application. In terms of the possible bias in a randomized controlled trial, the operators must be able to be proficient in the techniques studied [13], even all of the tracheal intubation in this study were performed by a specified anesthesiologist who were skilled in using the ultrasound and SOS in tracheal intubation. Finally, this study was performed in a single center on a relatively small number of patients and the results need to be confirmed by multicenter studies.

## Conclusions

This study validated that ultrasound-guided tracheal intubation was as effective as Shikani optical stylet-aided tracheal intubation in patients with anticipated normal airway.

## Data Availability

The datasets generated during and/or analyzed during the current study are available from the corresponding author on reasonable request.
